# Stopping clinical trials early for futility: retrospective analysis of several randomised clinical studies

**DOI:** 10.1186/1745-6215-12-S1-A53

**Published:** 2011-12-13

**Authors:** Mark Jitlal, Iftekhar Khan, Siow Ming Lee, Allan Hackshaw

**Affiliations:** 1 Cancer Research UK & UCL Cancer Trials Centre, University College London, London, W1T 4TJ, UK; 2 Department of Oncology, University College London Hospital, London, NW1 2PG, UK

## Background

Phase III clinical trials are generally large and expensive, so stopping early for futility is a potentially attractive approach. It could avoid using further patients and funds on an ineffective intervention. We aimed to see how well futility performs for clinical trials in practice.

## Materials and methods

We retrospectively applied a futility method to ten cancer trials, in which the final hazard ratios (HR) showed a large, moderate, or no treatment benefit. The target sample size was reached in all. The conditional power (CP) is the probability of obtaining a HR of a specified magnitude, which is statistically significant. Futility analyses were applied after 25, 50 and 75% of patients were recruited, or events observed. Two methods were used, assuming future data are consistent with either (i) the target HR, or (ii) the observed interim HR. Low CP suggests futility.

## Results

Futility analyses could stop some trials with no overall benefit, but not all, depending on the method used and timepoint. For example, after observing 50% of the target number of events, and assuming that future data is consistent with observed data, 4 out of 5 trials with no benefit could be stopped early (CP≤9%). Among 3 (of these 4) studies, trial duration could be reduced by 4-40 months, saving £44k-£385k. The other trial would have already finished accrual and hence no savings made. Of concern is that all 4 trials with moderate treatment effects could have stopped early at some point. For example, assuming that future data is consistent with the observed data, these trials could have stopped after 25% of patient accrual (CP≤9%). However, the final HRs for all four trials showed clinically worthwhile benefits.

## Conclusions

Appropriate application of futility methods can substantially shorten trial duration and reduce costs for trials which ultimately show no benefit. However, studies with moderate treatment effects could be stopped early, whilst some studies with no effect may not have been detected as such. Futility needs to be applied with great care to avoid missing a worthwhile treatment. We suggest several criteria for stopping a trial early: low CP (e.g. ≤15%); sufficient number of events; remaining patient accrual is likely to take several months; and lack of evidence of a benefit for important secondary endpoints and pre-defined subgroups.

